# Psychosocial and Functional Distress of Cancer Patients in A Tertiary Care Hospital: A Descriptive Cross-sectional Study

**DOI:** 10.31729/jnma.4491

**Published:** 2019-08-31

**Authors:** Guru Sharan Sah

**Affiliations:** 1 Department of Medical Oncology, B. P. Koirala Memorial Cancer Hospital, Bharatpur, Nepal

**Keywords:** *cancer*, *distress thermometer*, *Nepal*, *psychosocial stress*

## Abstract

**Introduction:**

Global burden of cancer is witnessing an exponential increase. Nepal is no exception. In the recent years, cancer care has seen a focus shift towards holistic healing. This includes screening and assessing for psychosocial distress, allowing health care providers to deliver timely psychological interventions. The goal of this study was to find the prevalence of psychosocial and functional impact of cancer diagnoses in Nepal.

**Methods:**

A descriptive cross-sectional study was carried out on 169 cancer patients attending outpatient department, day-care and in-patient department at B.P. Koirala Memorial Cancer Hospital, Nepal. National Comprehensive Cancer Network Distress Thermometer was used to evaluate spiritual/religious concerns, practical, family, emotional and physical issues and the distress score of these patients.

**Results:**

One-hundred and thirty eight (81.7%) of respondents had a Distress Thermometer score of ≥4. Distress Thermometer score of 7 was reported by the largest number of patients. Highest average Distress Thermometer scores were found in patients with hepatobiliary, head & neck and lung cancers. More than 50% of the patients reported to experience spiritual or religious concerns, fatigue, pain, worry and insurance or financial related concerns. Pain, sadness, worry and spiritual/religious concerns were significantly associated with distress levels. Sixty-two (36.7%) of respondents were in stage IV of cancer. Average Distress Thermometer score for patients in stage IV cancer was 5.69, the highest among all cancer stages. Ninety-six (56.8%) of the respondents were females, 73 (43.2%) were males. Gynaecological, haematological, gastrointestinal, head & neck and breast cancers were the top 5 cancer types.

**Conclusions:**

Cancer patients in Nepal have clinically significant psychosocial issues that directly impact on their distress.

## INTRODUCTION

Cancer incidence rate in Nepal is on the rise. By 2020, it is estimated to reach 38.5 and 41.4 per 100,000 for males and females respectively.^1^ Cancer diagnosis often precipitates a sense of doom.^2^ Patients are at risk of developing various physical and psychosocial conditions, which need to be identified and attended within the cancer care system.^3^ Approximately one third of cancer patients have physical, social and psychological problems and should receive focused psychosocial support.^4^

Worldwide, screening distress with the National Comprehensive Cancer Network's (NCCN) Distress Thermometer (DT) is recommended and routinely used to detect clinically significant ditress.^5,6,7^ In developing countries like Nepal, there is a paucity of awareness and data regarding the standardisation and use of DT in cancer patients and its impact.

We conducted this descriptive cross-sectional study to find the prevalence of the psychosocial and functional impact of diagnosis of cancer in patients in our country.

## METHODS

A descriptive cross-sectional study was conducted on cancer patients attending the out-patient department (OPD), day care and in-patient department of B.P. Koirala Memorial Cancer Hospital, Nepal. The duration of the study was six months, from July 2015 to December 2015. Distress score, spiritual/religious concerns, practical, family, emotional and physical issues of the patients were recorded and evaluated using NCCN Distress Thermometer (DT). DT is a questionnaire based quantitative scale of 0-10; a score of 10 representing the highest level of distress.

Sample size was calculated using the following formula

Sample size = Z^2^ ×pq/d^2^

    = 3.84 × 0.5×0.5/ (0.08)^2^

    = 150

p is the prevalence, 50%
q is the complement of p, i.e, q=100-p
d is allowable error, 8%

Z is the standard normal variate, which is 1.96 for 95% confidence interval.

Considering non-response rate of 10%, 165 was calculated and thus participants of 169 were included in the study. Inclusion criteria for this study was, clinically diagnosed and histopathologicaly proven malignancy. Convenience sampling was done. Verbal consent was obtained from patients participating in the study. Contents of DT were explained by treating physician and his team and the same recorded the response. All 169 patients’ responses were included in this analysis. However, responses of patients may result some elements of social desirability bias which is beyond our control in this study. Descriptive statistics was used to analyze qualitative and discrete variables. Statistical analysis was done using SPSS version 20.

## RESULTS

One hundred and sixty nine patients diagnosed with various cancers were evaluated in this study. Ninetysix (56.8%) of the respondents were females, while 73 (43.2%) were males. Frequency distribution of gender, smokers/non-smokers, alcoholics/non-alcoholics and respondents with knowledge on disease has been detailed (Table 1).

**Table 1:** Frequency distribution of demographic parameters like gender, smoking, alcoholism and respondent's knowledge on disease. N=169.

Category	Variable	n (%)
Gender	Male	73 (43.2%)
	Female	96 (56.8%)
Smoking	Smoker	54 (31.9%)
	Non-smoker	115 (68.1%)
Alcoholism	Alcoholic	47 (27.8%)
	Non-alcoholic	122 (72.2%)
Knowledge on Disease	With Knowledge	127 (75.2%)
	Without Knowledge	41 (24.3%)
	Data NA	1 (0.5%)

**Figure 1.**
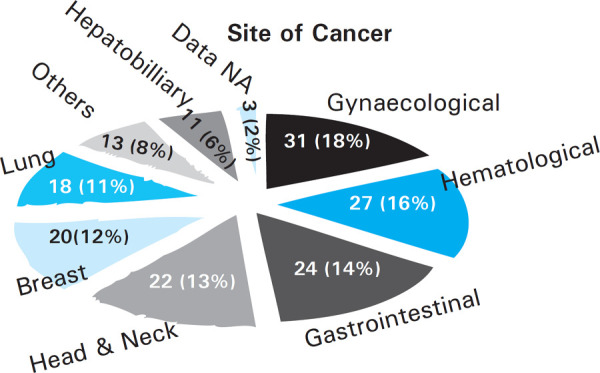
Distribution of type of cancer based on the site. N=169.

The cancer types (site of cancer) of these 169 respondents could be divided into 8 categories. Data for 3 patients were not available. Gynaecological (uterine, ovarian), hematological, gastrointestinal, head & neck and breast cancers were the top 5 cancer types in this study. The frequency distribution of these cancer types in the study has been illustrated (Figure 1).

We have categorised the cancer stages from the medical records of the respondents in this study into stage I-IV, at the time of diagnosis, as per the TNM classification of malignant tumors. Data for stage of cancer was not available for 25 patients. 62 (36.7%) of the respondents were found to be in stage IV of cancer, followed by 50 (29.6%) of the patients who were in stage II. The average DT score for patients in stage IV of cancer was 5.69, the highest among all the cancer stages for which data is available. Distribution of patients as per the stage of cancer and their corresponding mean DT score (Table 2).

**Table 2:** Stage of cancer and their corresponding mean DT score.

Stage of Cancer	n (%)	Mean DT Score
Stage I	11 (6.5%)	5.00
Stage II	50 (29.6%)	4.76
Stage III	21 (12.4%)	5.57
Stage IV	62 (36.7%)	5.69
Data NA	25 (14.8%)	5.88

**Figure 2.**
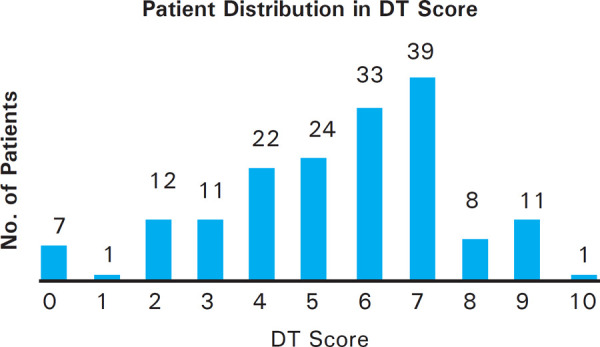
Patient distribution according to their reported DT score.

One hundred and thirty eight (81.7%) of the respondents evaluated in this study had a DT score of ≥4. DT score of 7 was reported by the largest number of patients in this study (39 patients, 23.1%). The distribution of patients according to their DT scores has been illustrated (Figure 2).

Among the type of cancer and their corresponding mean DT score, Hepatobilliary cancer has the highest score of 6.82, followed by Head & Neck at 6.41 and Lung cancer at 5.78. Data on the type of cancer was not available for 3 patients (Table 3).

**Table 3:** Patients with different types of cancer and the corresponding mean DT score.

Type of Cancer	No. of Patients	Mean DT Score
Hematological	27	4.89
Gynaecological	31	5.77
Head & Neck	22	6.41
Breast	20	5.15
Lung	18	5.78
Gastrointestinal	24	4.38
Others	13	4.23
Hepatobiliary	11	6.82
Data NA	3	5.33

**Figure 3.**
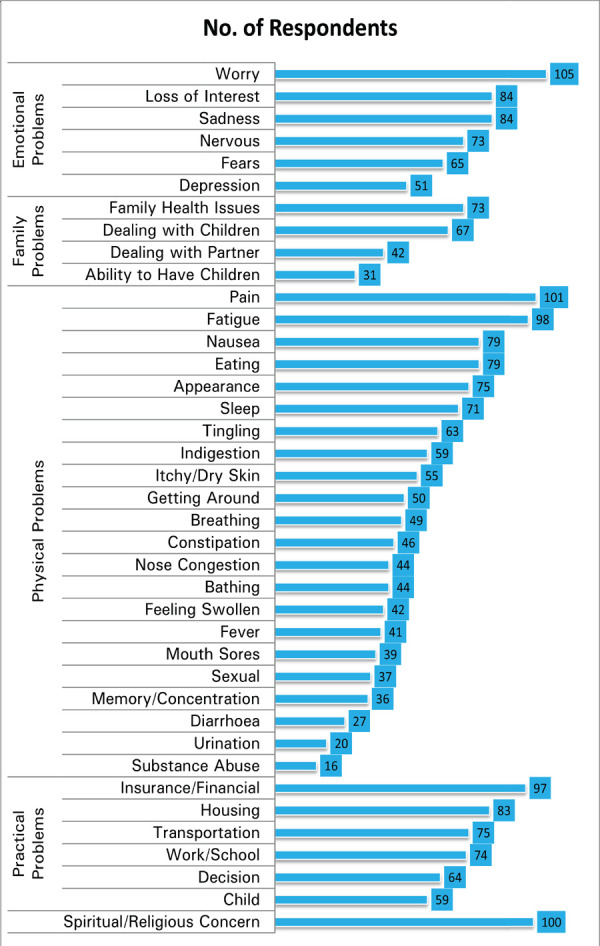
Distribution of respondents with respect to DT tool parameters.

The NCCN DT tool is categorised into 5 broad aspects – physical, practical, family, emotional and spiritual. More than 50% of the patients reported to experience spiritual or religious concerns 100 (59.2%), fatigue 98 (58%) and pain 101 (59.8%) in the category of physical problems, worry 105 (62.1%) in the category of emotional problems and insurance or financial related concerns 97 (57.4%) in the category of practical problems. Nearly half of the respondents also reported to experience loss of interest in usual activities 84 (49.7%) and sadness 84 (49.7%) in the category of emotional problems, eating 79 (46.7%) and nausea 79 (46.7%) related issues in the category of physical problems and housing related concerns 83 (49.1%) in the category of practical problems. Substance abuse 16 (9.5%) and changes in urination 20 (11.8%), both in the category of physical problems, seemed to be an area of concern for the least number of respondents in our study. Patient distribution with respect to all the parameters in DT tool has been illustrated (Figure 3).

## DISCUSSION

Most therapeutic modalities for cancer are associated with an array of morbidities ranging from minor and treatable to serious and potentially lethal. Cancer patients as well as long term cancer survivors report a decline in physical activity, dietary intake and overall quality of life.^8^ As a result, it is extremely important to understand the unique medical and psychosocial needs of cancer patients from diagnosis to long term survivorship and be aware of resources that can assist patients, caregivers, and health care providers in successfully managing the various phases of cancer survivorship.

The U.S. National Comprehensive Cancer Network (NCCN) has suggested that assessment of psychological distress in cancer patients should be added to the standard program of cancer treatment.^9,10^ The NCCN recommends that care providers distinguish a normal emotional reaction after cancer diagnosis from psychological distress, estimate the degree of potential distress in cancer patients, and identify patients with severe distress. Assessment of a high risk of psychological distress in patients allows health care providers to deliver timely psychological interventions and treatment.

In our study, we show a definite proof of the existence of psychosocial distress among cancer patients in Nepal. In fact, the psychosocial problems increase as the disease progress, as is evident from our finding that the mean DT score of 5.69 was the highest in stage IV cancer patients, whereas the mean DT scores were lower in stage I-III cancer patients. 138 (81.7%) of the respondents evaluated in our study had a DT score of ≥4. Ozalp et al showed that the DT cut-off score of 4 yielded the optimal combination of sensitivity and specificity for identifying clinically significant distress.^11^ Going by similar benchmark, more than 4/5^th^ of the respondents in our study were found to have clinically significant levels of psychosocial distress. In this study, DT score of 7 was reported by the largest group of patients 39 (23.1%). Shim et al reported that DT was found to be simple and effective screening instrument for detecting psychosocial distress in Korean cancer patients, and for identifying problems that warrant intervention.^12^

In our study, mean DT score of patients in all the cancer types showed clinically significant distress levels. However, the top 5 cancer types were Hepatobilliary cancer, followed by Head & Neck, Lung, Gynaecological and Breast cancers. Multiple studies have shown the devastating psychosocial impact of gynaecological and breast cancer diagnosis in women. Many patients develop symptoms of psychological distress such as anxiety, depression, fatigue, pain, difficulty concentrating, social isolation, sexuality concerns, and self-blame. In women, the loss of the symbols of femininity can result in low self-esteem, negative body image, false self-perception, social isolation and the development of communication or relationship problems with family members or friends.^13–20^ Buchmann et al reported high levels of psychosocial distress in head & neck cancer. Factors associated with increased distress level included a self-reported history of depression, family concerns, emotional concerns, and physical concerns.^21^ Stress, mainly of psychosocial nature, has been linked to the evolution of three important liver-related pathological entities - viral hepatitis, cirrhosis and hepatocellular carcinoma.^22^ Many authors have previously reported that lung malignancies are associated with high symptom burden, poorer prognosis, and stigmatization. Such factors increase psychological distress and negatively impact quality of life. Younger age and specific physical and psychosocial symptoms are predictive of clinically significant distress among lung cancer patients.^23–25^ Haematological malignancies have been shown to have devastating effects on the patients’ physical, emotional, psycho-sexual, educational and economic health. Improvement of therapies and infrastructure has resulted in such patients living longer. However significant proportion of these patient have been shown to have poor quality of life due to various physical and psychological consequences of the disease and the treatments.^26^ Overall, our findings are in sync with globally reported incidences of psychosocial distress in various cancer types.

In our study, more than 50% of the patients reported to experience spiritual or religious concerns, fatigue, pain, worry and insurance or financial related concerns. Nearly half of the respondents also reported to experience loss of interest in usual activities, sadness, eating problems, nausea and housing related concerns. Almost all of these psychosocial concerns are widely reported in literature for cancer patients. Synergistic relationship exists between depression and a broad array of physical symptoms in patients with advanced cancer.^27^ Declines in performance status and functional activity, problems in carrying on one's own daily activities, poor concentration, memory impairment, or altered sexuality are important in influencing the psychological response of cancer patients. The loss of certainty, the instability of one's own emotional status, like fear, anxiety, worry, and sadness, the need to depend on others, the reduction of self-esteem, the change of perspective about the future, and the threat of possible death are some examples of the multitude of emotional effects and experiences cancer patients have to deal with during the course of illness. Spiritual aspects of life like personal values, meaning ascribed to one's own life and existence, etc are important considerations for cancer patients. Social and interpersonal dimensions are also impacted by cancer and its treatment. Feelings of loneliness and abandonment, problems in returning to work, marginalisation, or even stigmatisation are common issues that cancer patients report.^28,29^ Quite expectedly, in our study we found that pain, sadness, worry and spiritual/religious concerns were significantly associated with distress levels as indicated by high DT scores.

World Health Organization (WHO) projections estimate the incidence of cancer to increase exponentially by the year 2030, with the annual number of new cases rising from 14.1 million in 2012 to 21.6 million in 2030 and deaths due to cancer rising from 8.8 million worldwide in 2015 to more than 12 million in 2030. At the same time, earlier diagnoses and improvement in cancer therapies have also led to an increase in survival that includes more than 300 million cancer survivors around the world. A broad implication of these figures involves the psychosocial impact of the disease, including emotional consequences, supportive care needs, and quality of life of cancer patients and their families.^30^ One third of the cancer patient population indicates an unmet need for psychosocial support or is actually using psychosocial services.^31,32^

In conclusion, ours is the first study in Nepal to show that cancer patients living in developing country like ours have definite psychosocial issues and that directly impact on the distress of these patients. Hence, these patients are in need of psychological, social and emotional support to cope up with their disease. We need to urgently acknowledge the findings of this study and create awareness as well as practice guidelines to provide holistic cancer care in our country.

The limitations of our study include relatively smaller sample size. Ours is a descriptive cross sectional study. Like any other studies based on psychometric tools, perception and social desirability bias of responders can not be completely ruled out. A larger, multicentric, longitudinal study design to record and analyse the trajectory of psychosocial response would further improve our understanding on this issue. Our findings are also limited to ethnic Nepalese and hence may not be representative of a culturally and demographically diverse population.

## CONCLUSIONS

Nearly more than half of cancer patients in Nepal have clinically significant psychosocial issues that directly impact on their distress. They need psychological, social and emotional support to deal with their disease. We need to develop country wide screening and assessment strategies to provide world class psychosocial support for patients diagnosed with cancer.

## Conflict of Interest:

**None.**
